# SjCRT, a recombinant *Schistosoma japonicum* calreticulin, induces maturation of dendritic cells and a Th1-polarized immune response in mice

**DOI:** 10.1186/s13071-017-2516-7

**Published:** 2017-11-13

**Authors:** Lizhen Ma, Dandan Li, Chunxiu Yuan, Xiangqian Zhang, Na Ta, Xiaochao Zhao, Yumei Li, Xingang Feng

**Affiliations:** 1 Shanghai Veterinary Research Institute, Chinese Academy of Agricultural Sciences, Key Laboratory of Animal Parasitology, Ministry of Agriculture of China, Shanghai, 200241 China; 2 College of Life and Environmental Sciences, Shanghai Normal University, Shanghai, 250014 China; 3 Jiangsu Co-innovation Center for Prevention and Control of Important Animal Infectious Diseases and Zoonoses, Yangzhou, Jiangsu Province 225009 China

**Keywords:** Calreticulin, *Schistosoma japonicum*, Dendritic cells, CD4+ T cells

## Abstract

**Background:**

It is well known that immunization of radiation-attenuated (RA) schistosoma cercariae or schistosomula can induce high levels of protective immunity against schistosoma cercariae reinfection in many animals. Many studies have shown that the Th1 cellular immune response is crucial for the protective effect elicited by RA schistosomula. However, the molecular mechanism of this strong protective immunity remains unclear.

**Methods:**

The expression profiles of *Schistosoma japonicum* calreticulin (SjCRT) in RA and normal schistosoma-derived cells were investigated by flow cytometry. The effect of recombinant SjCRT (rSjCRT) on mouse dendritic cells (DCs) was determined by FACS, ELISA and RT-PCR analysis. We also analyzed the effects of SjCRT on the activation of spleen cells from mice immunized with rSjCRT by detecting lymphocyte proliferation and the cytokine profiles of splenocytes.

**Results:**

We found that the expression level of SjCRT in the cells from RA larvae was significantly higher than that in cells from normal schistosomula at early stages of development (day 4). The results of effect of rSjCRT on mouse DCs showed that rSjCRT could induce phenotypic and functional maturation of DCs, and SjCRT bound to the surface of DCs through the CD91 receptor and could be engulfed by DCs. The results of activation of splenocytes from mice immunized with rSjCRT also demonstrate that rSjCRT can effectively stimulate the proliferative response of splenic lymphocytes, elicit splenocytes from immunized mice to secrete high levels of IFN-γ, TNF-α and IL-4, and activate CD4+ T cells to produce high levels of IFN-γ.

**Conclusion:**

SjCRT is one of the immunostimulatory molecules released from RA schistosomula cells, might play a crucial role in conferring a Th1-polarized immune response induced by RA cercariae/schistosomula in mice, and is a candidate molecule responsible for the high levels of protective immunity induced by RA schistosomula.

## Background

*Schistosoma japonicum* is a causative agent of “intestinal” and “hepatic” schistosomiasis and is still endemic in seven provinces of China [1]. In order to sustainably control schistosomiasis, there is an urgent need to develop prophylactic vaccines with high efficacy and safety. Previous studies have indicated that vaccination with radiation-attenuated (RA) larvae is highly effective in many experimental hosts. However, it is unfeasible to apply RA vaccines to hosts because they are either unsafe or unavailable due to the lack of resources of schistosome larvae. Therefore, researchers propose that a molecular vaccine against schistosomiasis might be developed according to the effective mechanisms of protective immunity induced by an RA vaccine [2, 3]. Further analysis of the mechanisms of protective immunity showed that irradiated lung-stage schistosomula (LS) were inducers of protective immunity, and LS was also shown to be the principal target of immunity in challenged animals [3]. This protective immunity is characterized by Th1-type immune responses, and is mainly mediated by CD4+ T-helper (Th) cells. However, the schistosome-derived cells/molecules responsible for the strong protective effects in the RA model remain unclear.

The concept of immunogenic cell death (ICD) was proposed to describe cellular mechanisms of anticancer immune responses, and accumulating experimental data indicate that immunogenic features of ICD are mediated by so-called ‘Damage-Associated Molecular Patterns’ (DAMPs), such as heat-shock protein (HSP), calreticulin (CRT), high mobility group protein B1 (HMGB1) and ATP [4]. Most of these molecules are intracellular molecules and have mainly nonimmunological functions within normal live cells, but they obtain immunostimulatory properties upon being exposed or released by damaged or dying cells [5]. DAMPs can exert their immunostimulatory effects when they are recognized by such receptors as membrane-bound/cytoplasmic pattern-recognition receptors, phagocytic/scavenger receptors and purinergic receptors. These DAMPs can mediate anticancer immunity because they can, together with cancer antigens, activate dendritic cells (DCs) and induce maturation of DCs, ultimately resulting in an adaptive immunity against cancer cells [4]. Among DAMPs, CRT has been more closely observed because Obeid et al. [6] showed that CRT exposure is a determining factor of immunogenicity of dying cancer cells. They found that anthracyclines can induce translocation of CRT to the surface of preapoptotic cells, and the immunogenicity of apoptotic cancerous cells can be suppressed by knockdown or blockade of CRT in mice. Therefore, immunogenic apoptosis associated with exposure of CRT on surface of the cell plays a key role in anticancer immunity.

In RA schistosome vaccine research, several studies have demonstrated differences in expression levels of immunogenic antigens between RA schistosomula and normal parasites. For example, Yang et al. [7] and Tian et al. [8] found that the expression level of *S. japonicum* heat-shock protein 70 (SjHSP70) derived from RA larvae is increased after RA treatment, and there is also evidence that the expression level of SjHSP70 on the cells from early RA schistosomula is significantly higher than that on cells from normal parasites [9]. Furthermore, in our preliminary study, a necrotic phenotype can be observed in *in vitro* cultured RA schistosomula. Therefore, we speculated that the immune responses induced by the RA schistosomula are qualitatively and/or quantitatively different from the immunity elicited by normal parasites. The increased immunogenicity of the RA larvae may be due to changes in the antigens exposed on the surface of the larvae and released into the extracellular milieu. These antigens include parasite-derived DAMPs, such as CRT, HSP70 and HMGB1. This situation might be similar to the aforementioned effector mechanism of anticancer immune responses induced by ICD. However, whether SjCRT is one of the main effectors of ICD or it might exert a key role in inducing high levels of innate and adaptive immune responses to an RA vaccine, remains to be identified. Thus in this study, we investigated the immunological function of SjCRT, including its expression profile in cells from RA and normal schistosomula, its recognition and engulfment by mouse dendritic cells, and the immunostimulatory effect of recombinant SjCRT on mouse DCs and splenic lymphocytes. We show that the expression level of SjCRT in early stage RA schistosomula was significantly higher than that in cells from normal parasites, and SjCRT could induce the maturation of DCs and facilitate a Th1-skewed immune response in mice.

## Methods

### Protein, parasites and animals

Recombinant SjCRT protein (rSjCRT) (GenBank: AAC00515) was obtained as described in Li et al. [10]. The limulus amebocyte lysate assay (LAL) was used to exclude LPS contamination of purified SjCRT protein (< 0.2 EU/ml, which has been referred to as endotoxin-free). The concentration of protein was detected by BCA (Shanghai Shenggong). Six- to eight-week-old BALB/c and C57BL/6 mice were purchased from the Shanghai Experimental Animal Center, Chinese Academy of Sciences. *Oncomelania hupensis* infected with *Schistosoma japonicum* was obtained from the Jiangsu Institute of Parasitic Diseases (Wuxi, China).

### Generation of immature DCs

Bone marrow cells were isolated from C57BL/6 mice. Then, 6 × 10^6^ cells/ml were cultured in complete RPMI supplemented with 2 ng/ml GM-CSF (Peprotech, Shanghai, China) and 1 ng/ml IL-4 (Peprotech) in 6-well plates at 37 °C, 5% CO_2_ for 3 days. On the 3rd day, 2 ml of the culture supernatant was carefully removed, and 3 ml of fresh complete culture medium was added to each well and continued to culture for another 2 days at 37 °C, 5% CO_2_. On day 5, the cells were collected, and immature DCs were isolated by using CD11c + MicroBeads (BD system, New Jersey, USA) according to the manufacturer’s protocol. These bmDCs were found to be > 95% CD11c + as detected by flow cytometry (Beckman FC-500MPL, California, USA).

### Preparation of polyclonal antibodies against SjCRT

Six- to eight-week-old female BALB/c mice were housed in a specific-pathogen-free (SPF) environment. Twenty mice were divided into two groups. One group was injected subcutaneously with 100 μg of rSjCRT emulsified in an equal volume of ISA206 adjuvant (Sigma-alorich, Shanghai, China), then mice were given two boosts with 50 μg rSjCRT emulsified with ISA206 adjuvant (Sigma) at 2 week intervals. At the same time, the other group was immunized with the same volume of PBS and ISA206 adjuvant as a control. Seven days after the third immunization, serum samples were collected from mice and stored at −80 °C until further analysis.

### Detection of the immunogenicity of SjCRT

To test the immunogenicity of SjCRT, purified rSjCRT and the crude adult worm antigens were loaded into 12% SDS-PAGE gels, separated, transferred onto 0.22 μm nitrocellulose membranes; the memebranes were blocked and washed as described in Duan et al. [9]. The membranes were then incubated in sera (diluted 1:100 in PBST) from mice immunized with rSjCRT, infected with *S. japonicum* and from SPF mice respectively, at 37 °C for 1 h. Next, membranes were washed three times with PBST and PBS respectively and incubated with 1:15,000-diluted IRDye800CW (LI-COR, Nebraska, USA)-conjugated goat anti-mouse IgG (eBioscience) in the dark for 40 min at room temperature. The membranes were washed three times and photographed with the Odyssey Infrared Imaging System.

### Detection of the expression of SjCRT from RA and normal schistosomula cells

The cells of RA and normal *S. japonicum* cercariae at different stages (4, 7, 10 or 14 days) were obtained as described in Duan et al. [9]. The schistosomula cells (2 × 10^6^) from the RA and normal groups were collected and washed with pre-cooled PBS, fixed in 4% phosphate-buffered paraformaldehyde for 40 min on ice, and washed twice with pre-cooled PBS. The cells were suspended in PBS containing 0.2% Triton-×100 and 5% rabbit serum on ice for 10 min and then washed twice with precooled PBS. Cells from the two groups were incubated with anti-SjCRT mouse serum (diluted 1:200 in PBST) at 4 °C overnight. Meanwhile, negative mouse serum is diluted 1:200 in PBST as control group. After washing twice with PBST, the cells were incubated with 1:2000-diluted Alexa-Fluor®-488-conjugated goat anti-mouse IgG (H + L) antibody in the dark for 40 min. After washing the cells with PBST for three times, they were re-suspended in 400 μl of pre-cooled PBS and analyzed by flow cytometry.

### Detection of the effect of rSjCRT on immature DCs

Immature 5-day DCs were obtained according to the above methods. The immature DCs (2 × 10^6^ cells/each well of a 48-well plate) were stimulated with 50 μg/ml rSjCRT, 50 μl PBS and 50 ng/ml LPS at 37 °C under 5% CO_2_ for 48 h. Then, DCs were harvested and stained with PE-conjugated mAbs to CD40 and PE Cy7-conjugated mAbs to CD86 (BD Systems, New Jersey, USA) at 4 °C in the dark for 30 min, and these cells were washed twice and resuspended in 400 μl of PBS and analyzed by flow cytometry. The supernatant was collected and IL-4, IL-10, TNF-α and IFN-γ were detected using enzyme-linked immunosorbent assay (ELISA) kits (R&D Systems, Minnesota, USA). The above stimulated DCs were harvested, and the expression of CCR7 and CXCR4 in DCs was assessed by RT-PCR and flow cytometric analysis. The primers used were 5′-CAT GGA CCC AGG TGT GCT TCT-3′ and 5′-GTC AGT ATC ACC AGC CCG TT-3′ for CCR7 and 5′-ATG GAA CCG ATC AGT GTG AGT-3′ and 5′-TTG CCG ACT ATG CCA GTC AA-3′ for CXCR4. The harvested DCs were stained with FITC-conjugated mAbs to CCR7 and PE-conjugated mAbs to CXCR4 (BD Systems) at 4 °C in the dark for 30 min, and these cells were washed twice and resuspended in 400 μl of PBS and then analyzed by flow cytometry.

### Detection of polarization of CD4+ T cells by rSjCRT-pulsed mouse DCs

Splenocyte suspensions were prepared from 6 to 8 week-old C57BL/6 mice according to the method as described by Duan et al. [9]. CD4+ T cells from the splenocyte suspensions were isolated using high-gradient magnetic cell sorting (MACS, BD Systems) according to the manufacturer’s protocol. DCs were pulsed with 50 μg/ml rSjCRT, PBS or 50 ng/ml LPS at 37 °C under 5% CO_2_ for 48 h, and these DCs were then harvested and washed twice with RPMI 1640. The aforementioned CD4+ T cells were divided into 4 groups: Control group (1 × 10^6^ CD4+ T cells and 1 × 10^5^ DCs were co-cultured in the same well of a 48-well plate), ConA group (1 × 10^6^ CD4+ T cells plus 5 μg ConA), LPS group (1 × 10^6^ CD4+ T cells and 1 × 10^5^ DCs pulsed with LPS) and SjCRT group (1 × 10^6^ CD4+ T cells and 1 × 10^5^ DCs stimulated with rSjCRT). These 4 groups were incubated in the dark at 37 °C under 5% CO_2_ for 7 days. Six hours before the cells were harvested, PMA (0.5 μg/ml), ionomycin (1 μg/ml) and monensin (3.5 μg/ml) were added to each well. After being washed twice with cell staining buffer (CSB), the cells were stained in 200 μl of CSB containing 2 μl of PE-Cy5-conjugated rat anti-mouse CD4 antibody at 4 °C in the dark for 30 min. After washing twice with CSB, the cells were incubated in 800 μl of 4% paraformaldehyde on ice in the dark for 15 min. The cells were then washed twice with CSB, and resuspended with 100 μl 1% Triton-×100 on ice in the dark for 20 min. After washing again twice with CSB, the cells were stained with FITC-conjugated rat anti-mouse IFN-γ antibody and PE-conjugated rat anti-mouse IL-4 antibody at 4 °C in the dark for 30 min. Cells were harvested and analyzed with flow cytometry.

### Observation of engulfment of rSjCRT and detection of fluorescent rSjCRT binding with CD91

rSjCRT was conjugated to fluorescein isolthiocyanate (FITC) using a FITC conjugation kit (Abcam, Shanghai, China). Immature 5-day DCs were incubated with 20 μg/ml FITC-SjCRT in PBS containing 1% skim milk powder in the dark for 20 min at 4 °C. These DCs were washed twice with pre-cooled PBS. The pellet was then resuspended in 50 μl precooled PBS and the suspension was mounted onto a precooled glass slide and visualized by a confocal microscope. The competitive binding assay was performed as follows: immature 5-day DCs were divided into several groups, and incubated in 2% paraformaldehyde on ice for 10 min. The cells were washed twice and incubated with 0 μg/ml rSjCRT, 20 μg/ml rSjCRT, 100 μg/ml SjCRT and 20 μg/ml α2-Macroglobulin (ligand of CD91) respectively at 4 °C for 30 min and then further incubated with 2 μl of FITC-SjCRT at 4 °C in the dark for 30 min. The cells were washed twice and resuspended in 400 μl of PBS and analyzed by flow cytometry.

### Lymphocyte proliferation and cytokine analysis of splenocytes in mice immunized with rSjCRT

In order to perform the lymphocyte proliferation assay, three mice of each group were selected. Seven days after the final immunization as described above, spleens were removed from the mice using scissors and forceps. The spleens were washed with cold RPMI 1640 medium (HyClone) and single-cell suspensions were obtained by passing them through a cell strainer. The suspensions were centrifuged at 1800× *rpm* for 5 min at 4 °C. Removing the supernatant, the precipitation was incubated with 2 ml of red blood cell lysis buffer (TIANGEN) for 2 min to lyse the erythrocytes, and the reaction was stopped by adding 8 ml of RPMI 1640 medium (HyClone). After washing twice with RPMI 1640 medium, the cells were resuspended in RPMI 1640 medium supplemented with 10% BSA to a final density of 1 × 10^6^ cells/ml. Then, 200 μl of the resuspension was seeded into a 96-well plate containing 5 μg of ConA or 1 μg of rSjCRT. As the control group, the cells from the PBS group containing the same volume of ConA or PBS. The cells were cultured at 37 °C under 5% CO_2_ in the dark for 72 h. After incubation for 68 h, 100 μl of the culture supernatants of each well were collected for cytokine assay. Then, 50 μl of MTT (5 mg/ml) was added to each well, and the cells were incubated for another 6 h. The plates were centrifuged at 1000× *rpm* for 10 min. The supernatants were gently removed, 100 μl of DMSO was added to each well, and plates were incubated at 37 °C in the dark for 5 min. The absorbance was analyzed at 570 nm by a microplate reader (Thermo Multiskan MK3, Waltham, Massachusetts, USA). The values of stimulation index (SI) were calculated as follows:

$$ \mathrm{SI}=\frac{\mathrm{Experimental}\  \mathrm{cells}\kern0.5em \mathrm{A}\ \mathrm{value}-\mathrm{control}\ \mathrm{A}\ \mathrm{value}}{\mathrm{Black}\  \mathrm{control}\  \mathrm{cells}\ \mathrm{A}\ \mathrm{value}-\mathrm{control}\ \mathrm{A}\ \mathrm{value}} $$

The levels of IL-4, IL-10, TNF-α and IFN-γ were detected using ELISA kits (R&D Systems).

### Detection of the ratio of CD4+/CD8+ T cells from spleens in mice immunized with rSjCRT

On day 10 after the final immunization, three mice from each group were euthanized to obtain the spleens. Spleen single-cell suspensions were collected as above. The cells were centrifuged at 1500× *rpm* for 5 min at 4 °C, the supernatant was removed and the pellet was resuspended in 200 μl of CSB. Then, the cells were stained with 2 μl of PE-Cy7-conjugated rat anti-mouse CD3 antibody, 2 μl of PE-conjugated rat anti-mouse CD4 antibody and 2 μl of PE-Cy5-conjugated rat anti-mouse CD8 at 4 °C in the dark for 30 min. The cells were washed twice with cold PBS, and were resuspended in 400 μl of cold PBS and analyzed by flow cytometry.

### Intracellular cytokines assay of CD4+ T cells from spleens in mice immunized with rSjCRT

On day 10 after the final immunization described above, four mice from each group were euthanized to obtain the splenic lymphocytes. The cells were counted and cultured in 48-well plates at 1 × 10^7^ cells/ml. Then, 50 μg of rSjCRT was added to cells from rSjCRT-immunized mice to restimulate the cells, and the same volume of PBS was added to cells from the PBS-treated mice as controls. When cultured for 68 h, PMA (0.5 μg/ml), ionomycin (1 μg/ml) and monensin (3.5 μg/ml) were added to each well and incubated for another 6 h. The cells were harvested and washed twice with CBS. Cells were then resuspended in 200 μl of CBS and 2 μl of PE-Cy5-conjugated rat anti-mouse CD4 antibody was added. The cells were incubated at 4 °C in the dark for 30 min, washed twice with CBS and incubated in 800 μl 4% paraformaldehyde on ice in the dark for 15 min. The cells were washed twice with CBS and resuspended with 100 μl 1% Triton-×100 on ice in the dark for 20 min. After washing twice with CBS, the cells were incubated with FITC-conjugated rat anti-mouse IFN-γ, PE-conjugated rat anti-mouse IL-4 antibody at 4 °C in the dark for 30 min. The cells were harvested, washed twice, and analyzed using a flow cytometry.

### Statistical analyses

Statistical analyses were completed using GraphPad Prism 5 software. The Student’s test for analysis of data from different groups and analysis of variance (ANOVA) was used to test the differences among groups. The flow cytometry results were analyzed by FlowJo. The results were considered significant at a *P*- value of < 0.05.

## Results

### Expression of SjCRT and its identification by immunoblotting

**Fig. 1:**
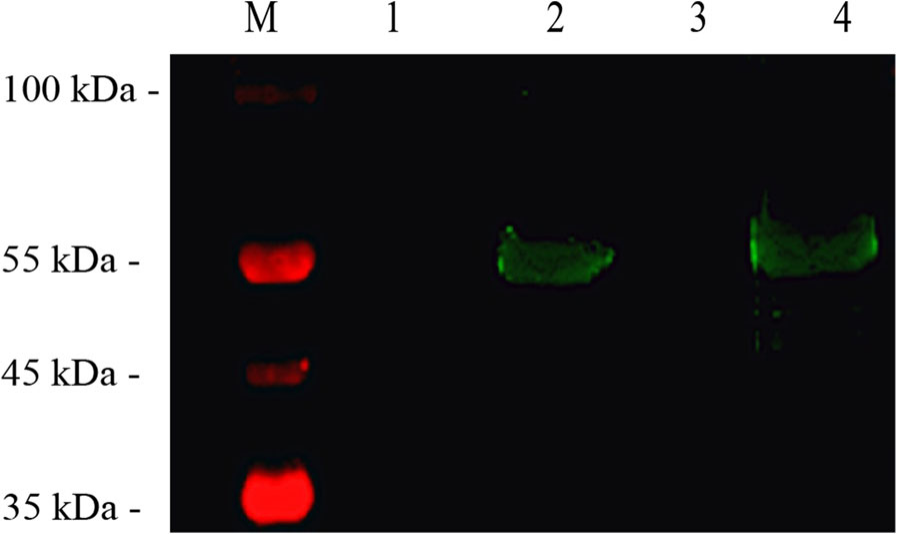
Analysis of the reactogenicity, antigenicity and immunogenicity of rSjCRT. Lane M: protein molecular weight marker; Lane 1: rSjCRT is recognized by the negative serum of SPF mice; Lane 2: rSjCRT is recognized by sera of mice infected with *Schistosoma japonicum*; Lane 3: Negative serum from SPF mice recognizes the crude worm antigen of *Schistosoma japonicum*; Lane 4: rSjCRT antiserum recognizes the crude adult worm antigen of *Schistosoma japonicum*

The full length SjCRT nucleotide sequence amplified from the *S. japonicum* cDNA is comprised of 1188 nucleotides, has no signal peptides, and encodes a 396-amino acid protein with a predicted molecular mass of approximately 55 kDa. We constructed a plasmid of pET-28a-SjCRT and obtained a recombinant SjCRT protein. The results of immunoblotting demonstrated that the recombinant protein can be recognized by the serum of mice infected with *S. japonicum* (Fig. 1, Lane 2), the antiserum from mice immunized with rSjCRT also reacted with a crude adult worm antigen of 55 kDa (Fig. 1, Lane 4), suggesting that rSjCRT has good antigenicity and that sera against rSjCRT can be used to determine the expression profile of SjCRT in the cells from normal and RA schistosomula.

### Expression profile of SjCRT in the cells from RA and normal schistosomula cultured *in vitro* at different time points

**Fig. 2:**
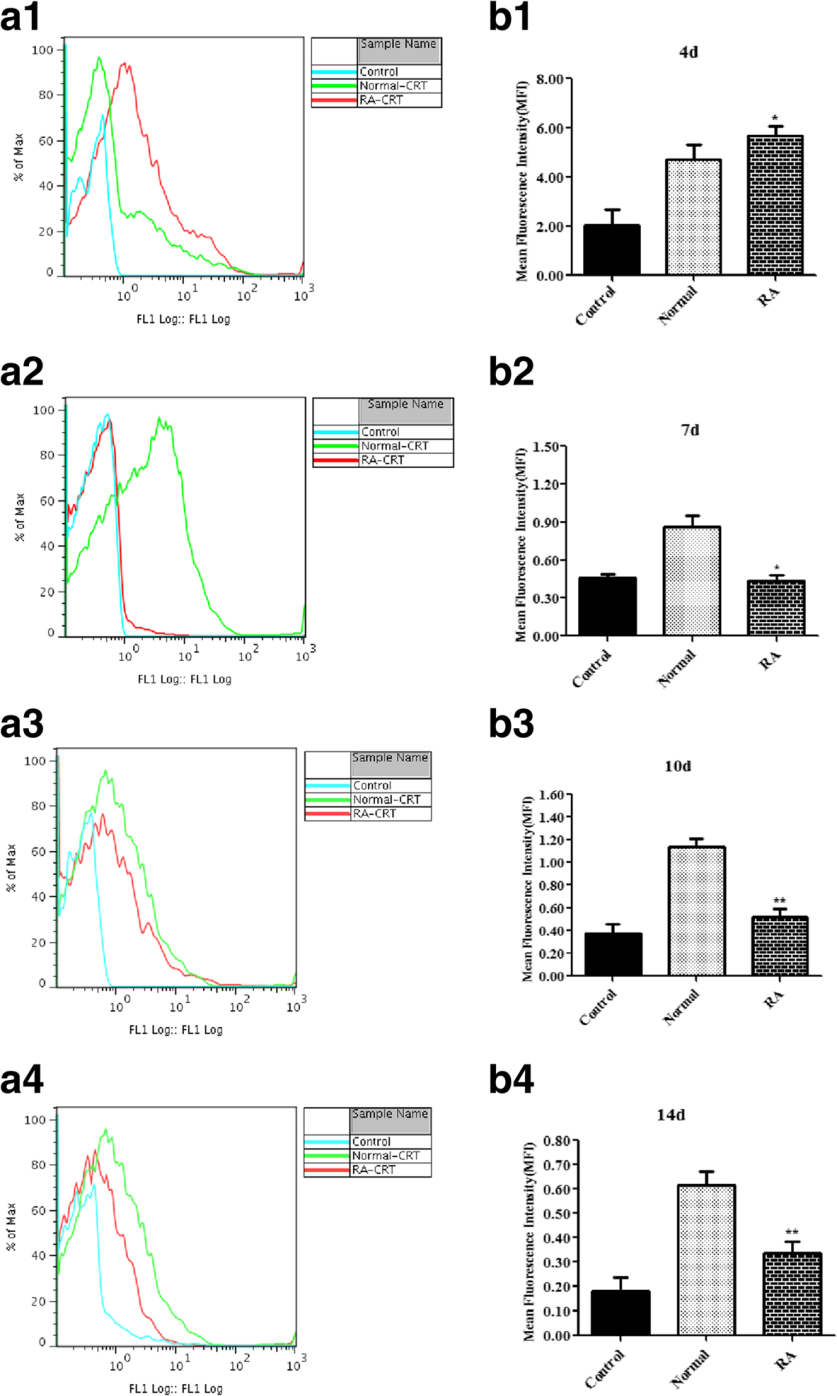
SjCRT expression from RA and normal schistosomula at different stages. Cercariae were exposed or not exposed to a UV source (254 nm) at an intensity of 400 μW/cm^2^ for 1 min and the tails were detached before culture for 4, 7, 10 or 14 days. Schistosomula were digested into single cells and the cytomembrane was ruptured with 0.2% Triton-×100 on ice for 10 min. Then the cells were collected to determine the expression of SjCRT by flow cytometry. Data illustrating representative experiments and derived from triplicate experiments are shown as a graph (**a1-a4**) and mean fluorescence intensities (**b1-b4**) showing the means ± SD from 4 samples, representative of two separate experiments. Statistically significant differences compared with the same stage of normal groups are shown as *P < 0.05 or **P < 0.01 (**b1**: *t*_(3)_ = 4.571, *P* = 0.014; **b2**: *t*_(3)_ = 5.537, *P* = 0.0311; **b3**: *t*_(3)_ = 9.415, *P* = 0.0025; **b4**: *t*_(3)_ = 6.629, *P* = 0.0070)

To investigate whether there are differences in the expression profiles of SjCRT between RA and normal schistosomula, SjCRT expression from RA and normal schistosomula at different points in time were detected by flow cytometry. The expression level (mean fluorescence intensity, MFI) of SjCRT from RA schistosomula-derived cells was significantly higher than that of normal larvae on day 4 (Fig. 2 a1, b1 b1: *t*_(3)_ = 4.571, *P* = 0.014) after treatment. Meanwhile, on days 7, 10 and 14 after treatment, the expression of SjCRT from RA schistosomula was significantly lower than that from normal parasites (Fig. 2 a2–4, b2–4 b2: *t*_(3)_ = 5.537, *P* = 0.0311; b3: *t*_(3)_ = 9.415, *P* = 0.0025; b4: *t*_(3)_ = 6.629, *P* = 0.0070). These data suggest that SjCRT has higher expression levels in cells from very early stages of RA schistosomula.

### rSjCRT can be engulfed by DCs through CD91 and induce DC maturation

**Fig. 3:**
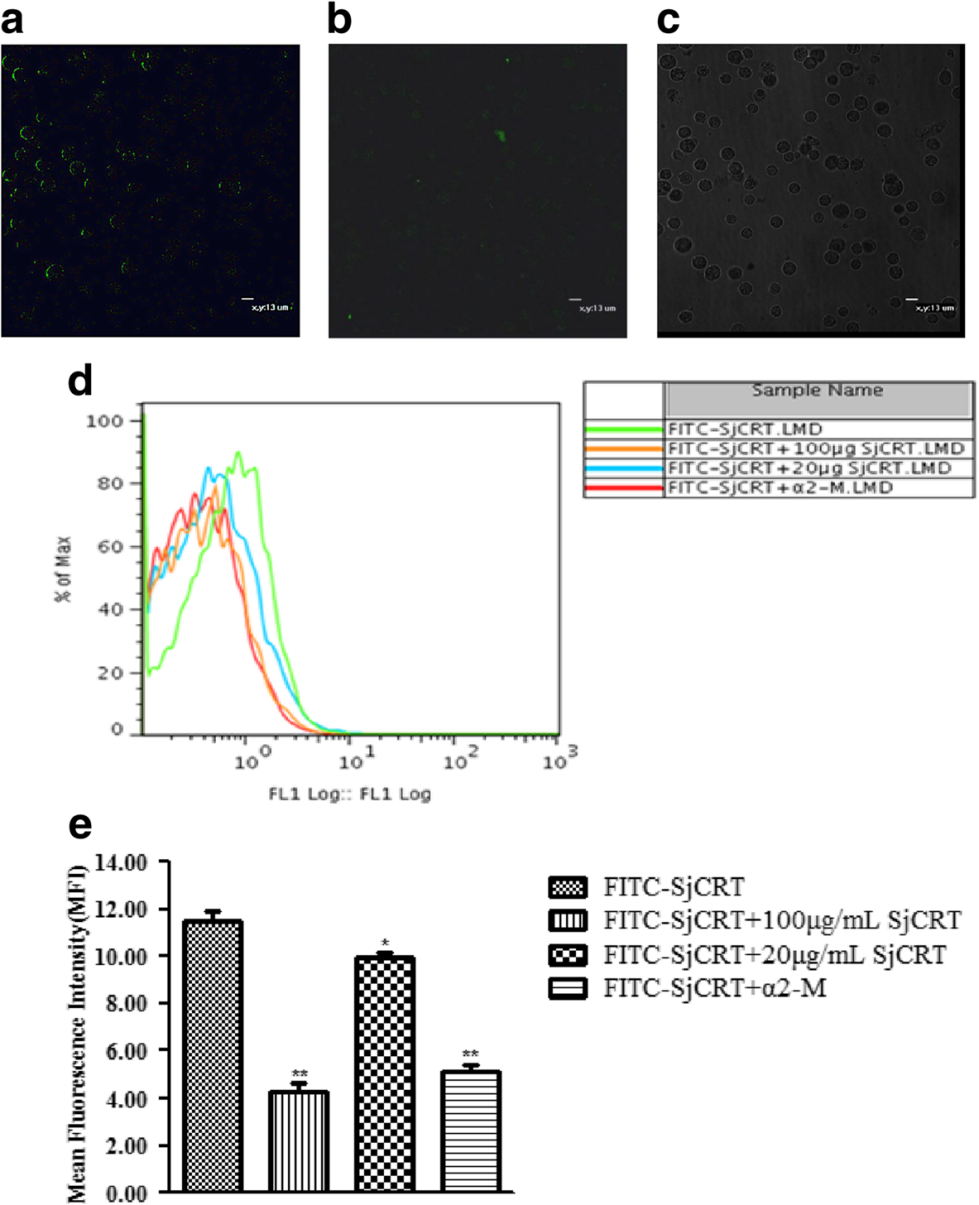
FITC-SjCRT binds to DCs and can be engulfed by DCs through CD91. **a** FITC-SjCRT binds to the surface of DCs at 4 °C. **b** FITC-SjCRT is endocytosed by DCs at room temperature. **c** Control of the same visual field. DCs fixed in paraformaldehyde were incubated with 20 μg/ml unlabeled SjCRT, 100 μg/ml unlabeled SjCRT and α2-M at 4 °C for 30 min, and 2 μl FITC-SjCRT (2 μg/ml the final concentration) was then added at 4 °C for 20 min. Then, cells were washed twice with precooled PBS, resuspended in 300 μl precooled PBS and analyzed by flow cytometry. Data illustrating representative experiments derived from quadruplicate experiments (**d**) and mean fluorescence intensities (**e**) are shown. Statistically significant differences compared with the control (FITC-SjCRT group) group are shown as *P < 0.05 or **P < 0.01 (100 μg/ml SjCRT: *t*_(3)_ = 15.66, *P* = 0.0041; 20 μg/ml SjCRT: *t*_(3)_ = 4.038, *P* = 0.0482; α2-M *t*_(3)_ = 15.93, *P* = 0.0039)

Previous studies have revealed that CRT can exert its effects on anticancer immunity because it can be engulfed by DCs and induce maturation of DCs [11]. Therefore, we hypothesized that SjCRT may have similar immunobiological properties in antischistosomes immunity induced by RA schistosomula. To this end, we first investigated whether SjCRT could be engulfed by DCs. As described in Methods, FITC-labeled rSjCRT (rSjCRT-FITC) was incubated with immature mouse bone marrow-derived DCs, and the cells were observed under laser confocal microscopy. The results showed that green fluorescence could be observed both on the surface of the cells at 4 °C (Fig. 3a) and in the cytoplasm at room temperature (Fig. 3b), indicating that rSjCRT-FITC could bind to the surface of DCs and be engulfed by DCs. According to previous studies [12], CD91 is a common receptor for heat shock proteins hsp70, hsp90, CRT and gp96. Therefore, we determined whether SjCRT is also taken up by DCs through CD91. We performed a competitive binding test by using unlabeled rSjCRT and α2-macroglobulin (α2-M, a known ligand for the CD91 receptor) to evaluate decreases in rSjCRT-FITC uptake by DCs. We found that 100 μg/ml SjCRT (*t*_(3)_ = 15.66, *P* = 0.0041), 20 μg/ml SjCRT (*t*_(3)_ = 4.038, *P* = 0.0482) and α2-M (*t*_(3)_ = 15.93, *P* = 0.0039) can significantly inhibit binding of rSjCRT-FITC to DCs as shown in Fig. 3c, d, indicating that both rSjCRT and α2-M have a common receptor of CD91 on DCs.

**Fig. 4:**
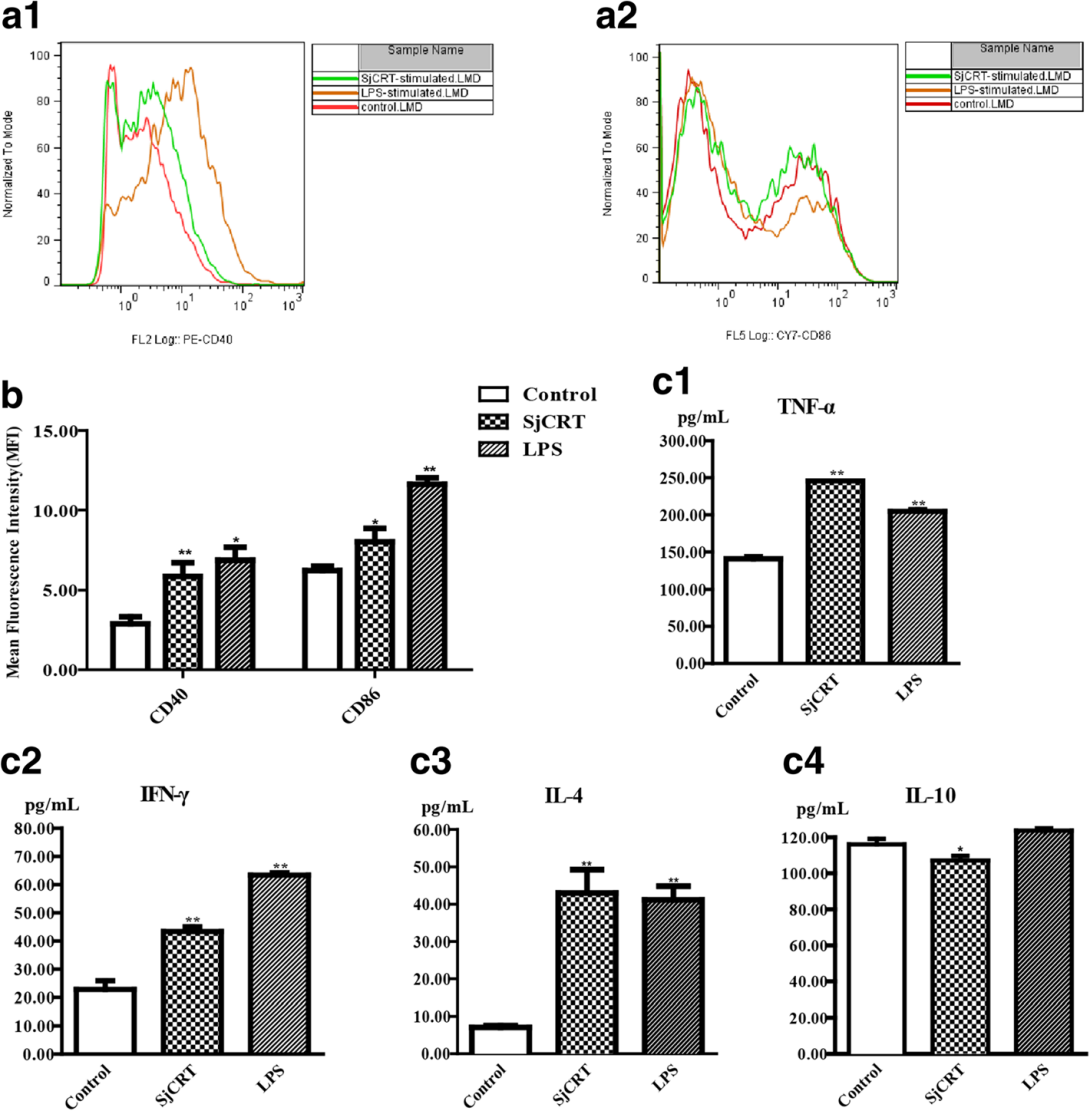
Phenotypic and functional maturation of DCs induced by rSjCRT protein. DCs were treated with rSjCRT, LPS or PBS for 48 h, and cells were then collected for FACS analysis of CD40 and CD86 expression. CD40: LPS *t*_(3)_ = 5.506, *P* = 0.0314; SjCRT: *t*_(3)_ = 11.93, *P* = 0.0070; CD86: LPS *t*_(3)_ = 14.73, *P* = 0.0046; SjCRT: *t*_(3)_ = 3.486, *P* = 0.0252. Supernatants were collected for ELISA analysis of IL-4, IL-10, TNF-α and IFN-γ secretion. PBS was the negative control, and LPS was the positive control. Data illustrating representative experiments derived from triplicate experiments (**a1-a2**) and mean fluorescence intensities (**b**) are shown. The production of cytokines is shown (**c1-c4**) TNF-α: LPS *t*_(5)_ = 29.13, *P* = 0.0012, SjCRT: *t*_(5)_ = 65.63, *P* = 0.0002; IFN-γ: LPS *t*_(5)_ = 25.48, *P* < 0.0001, SjCRT *t*_(5)_ = 10.70, *P* = 0.0017; IL-4: LPS *t*_(5)_ = 23.78, *P* < 0.0001, SjCRT *t*_(5)_ = 13.45, *P* = 0.0001; IL-10: LPS *t*_(5)_ = 3.472 *P* = 0.0403, SjCRT *t*_(5)_ = 3.294, *P* = 0.0460. Statistically significant differences compared with the negative control group are shown as *P < 0.05 or **P < 0.01

Since rSjCRT protein can be taken up by DCs, we further asked whether it can elicit maturation of DCs. In our previous study, we had indicated that rSjCRT can upregulate the expression of CD80 and MHC-II on mouse bone marrow-derived DCs (mDCs). In the present study, the expression of CD40 and CD86 on the surface of DCs was detected by flow cytometry. As shown in Fig. 4 a1-a2, b, rSjCRT can induce upregulation of CD40 (*t*_(3)_ = 11.93, *P* = 0.0070) and CD86 expression on mDCs (*t*_(3)_ = 3.486, *P* = 0.0252), indicating that rSjCRT can elicit the phenotypic maturation of mDCs. To determine whether rSjCRT can activate DCs and induce the functional maturation of DCs, the supernatants of 5-day DCs stimulated with rSjCRT were collected and analyzed for TNF-α, IFN-γ, IL-4 and IL-10 secretion by ELISA. We found that DCs stimulated with rSjCRT secreted significantly higher amounts of TNF-α (*t*_(5)_ = 65.63, *P* = 0.0002), IFN-γ (*t*_(5)_ = 10.70, *P* = 0.0017) and IL-4 (*t*_(5)_ = 13.45, *P* < 0.0001) than those in the control group (Fig. 4 c1-c3). In contrast, rSjCRT could significantly suppress the secretion of IL-10 by DCs (*t*_(5)_ = 7.10, *P* = 0.04) compared with the control group (Fig. 4 c4). The observed cytokine profile indicates that rSjCRT can induce functional maturation of DCs and suggests a Th1 bias, as TNF-α and IFN-γ typically induce Th1 polarization.

**Fig. 5:**
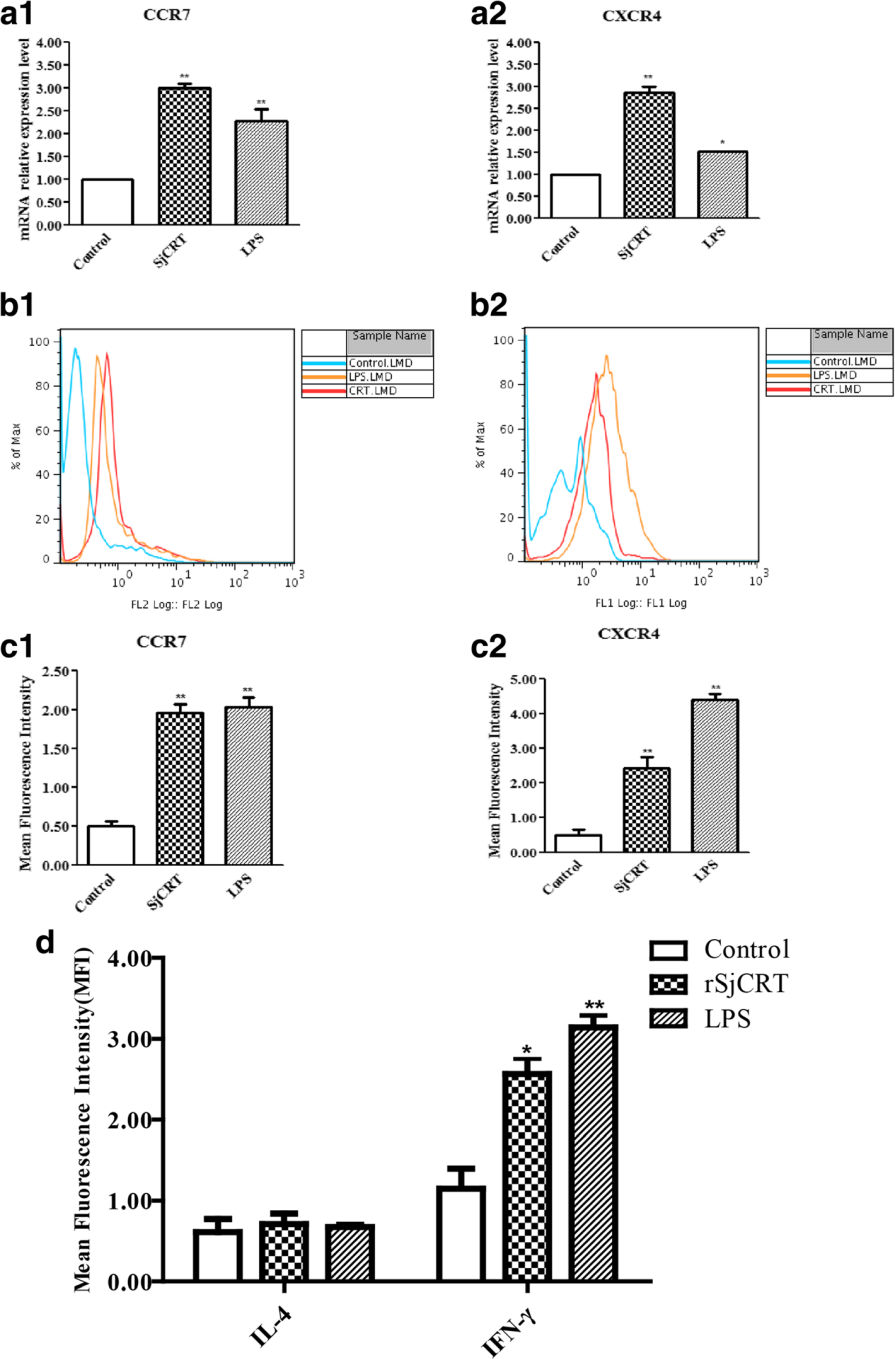
SjCRT stimulates DCs to express the chemokine receptors CCR7 and CXCR4 and promotes CD4^+^ T cells to secrete Th1-type cytokines. DCs were stimulated with rSjCRT, LPS or PBS for 48 h. Cells were then harvested for analysis of CCR7 and CXCR4 expression by quantitative RT-PCR (**a1**-**a2**) CCR7: LPS *t*_(4)_ = 18.70, *P* = 0.0079, SjCRT *t*_(4)_ = 37.52, *P* = 0.0007; CXCR4: LPS *t*_(4)_ = 8.30, *P* = 0.02, SjCRT *t*_(4)_ = 23.05, *P* = 0.0019; and FACS (**b1**-**b2**, **c1**-**c2**) CCR7: LPS *t*_(3)_ = 14.54 *P* = 0.0047, SjCRT *t*_(3)_ = 14.39, *P* = 0.0048; CXCR4: LPS *t*_(3)_ = 111.7, *P* < 0.0001, SjCRT *t*_(3)_ = 9.931 *P* = 0.0100. Immature 5-day DCs were stimulated with 50 μg/ml SjCRT, 50 μl PBS or 50 ng/ml LPS at 37 °C under 5% CO_2_ for 48 h. The PBS group was used as the negative control, and the LPS group was the positive control. Isolated CD4^+^ T cells from the splenocytes were incubated with different groups of DCs at 37 °C under 5% CO_2_ for 7 days. The production of intracellular cytokines IL-4 and IFN-γ by CD4^+^ T cells was measured by FACS (**d**) IL-4: LPS *t*_(3)_ = 2.458, *P* = 0.0394, SjCRT *t*_(3)_ = 2.693, *P* = 0.0742; IFN-γ: LPS *t*_(4)_ = 10.90, *P* = 0.0017, SjCRT *t*_(3)_ = 3.809, *P* = 0.0318. Statistically significant differences compared with the negative control group are shown as *P < 0.05 or **P < 0.01

It has been shown [13] that, under the condition of elevated levels of co-stimulatory molecules (CD40, CD80 and CD86) and increased levels of proinflammatory cytokines (TNF-α, IL-6), DCs that undertake immunogenic phagocytosis can present antigens to CD4+ T cells. These activated DCs can further activate Th1 cells, namely, IFN-γ-producing CD4+ T cells. To test this possibility, rSjCRT-pulsed mDCs were incubated with naive CD4+ T cells, and production of the intracellular cytokines IFN-γ and IL-4 by CD4+ T cells was then detected by flow cytometry (Fig. 5d). CD4+ T cells incubated with rSjCRT-pulsed mDCs produced significantly higher levels of IFN-γ (*t*_(3)_ = 3.809, *P* = 0.0318) than those incubated with control mDCs. Meanwhile, there was no significant difference in the levels of IL-4 (*t*_(3)_ = 2.693, *P* = 0.0742) between the aforementioned groups of CD4+ T cells. These data indicate that mDCs pulsed with rSjCRT can facilitate differentiation of Th1 cells *in vitro*.

It was reported that the expression of chemokine receptors CCR7 and CXCR4 is induced during DC maturation [14, 15], and those chemokine receptors might direct the migration of DCs to the draining lymph nodes (LNs). Therefore, we assayed the expression levels of CCR7 and CXCR4 in DCs stimulated with rSjCRT using RT-PCR and flow cytometric analysis. We found that, after exposure to rSjCRT and LPS stimuli, DCs can upregulate expression of CCR7 and CXCR4 (Fig. 5 a1-a2; b1-b2; c1-c2), further suggesting that rSjCRT can induce functional maturation of DCs.

### rSjCRT promotes lymphocyte proliferation and induces a Th1-biased immune response in mice immunized with rSjCRT

**Fig. 6:**
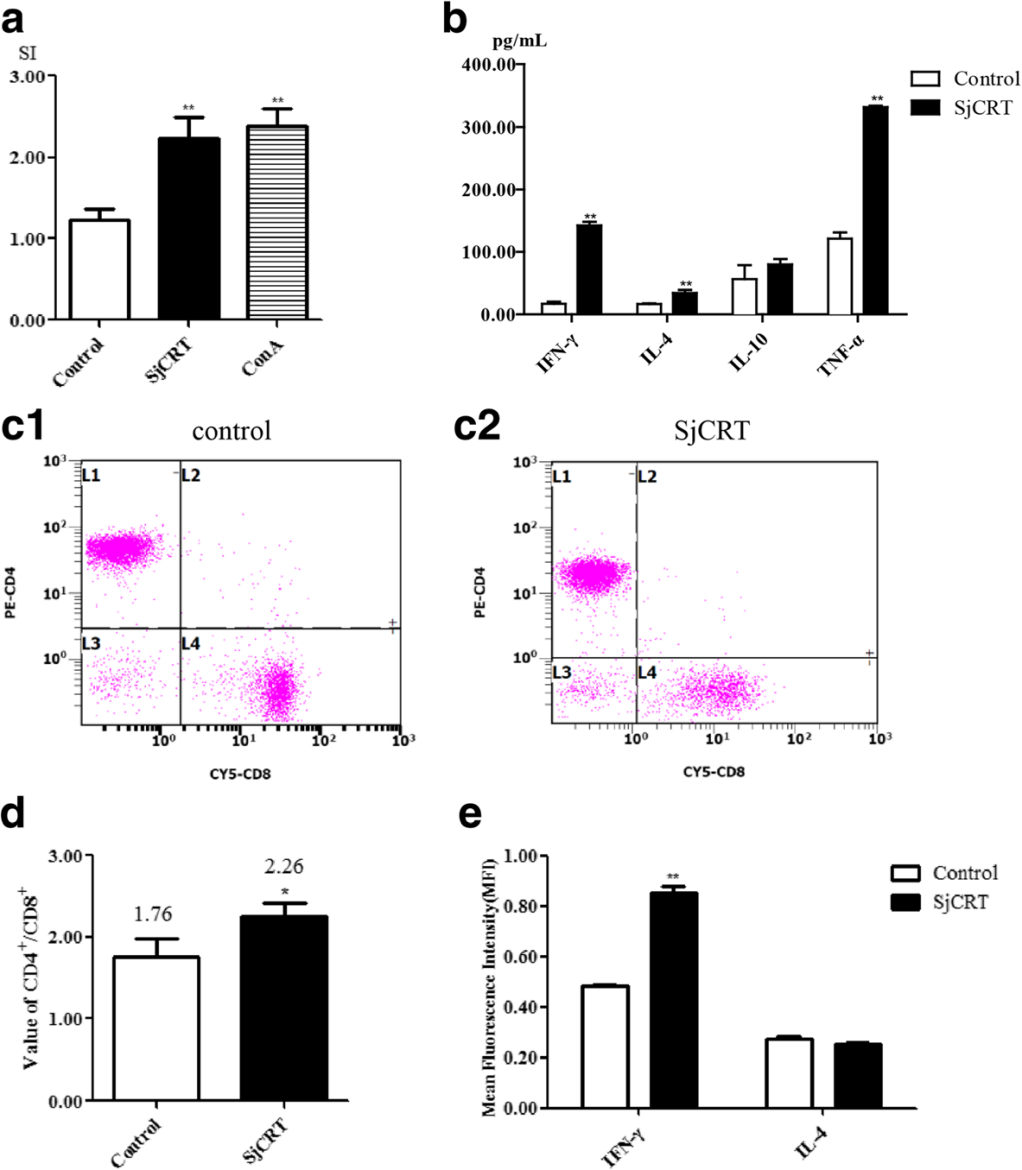
Effect of rSjCRT on lymphocytes in mice. Splenocytes from immunized mice were re-stimulated with rSjCRT or PBS at 37 °C under 5% CO_2_ for 72 h. Splenocyte proliferation was assayed by MTT (**a**) SjCRT: *t*_(3)_ = 20.44, *P* = 0.0003; ConA: *t*_(3)_ = 6.888, *P* = 0.0063. The supernatants were collected, and the concentrations of IFN-γ, IL-4, IL-10 and TNF-α were measured by ELISA (**b**) IFN-γ: *t*_(3)_ = 29.63, *P* < 0.0001; IL-4: *t*_(3)_ = 6.295, *P* = 0.0081; IL-10: *t*_(3)_ = 1.510, *P* = 0.2283; TNF-α: *t*_(3)_ = 41.39, *P* < 0.0001. The re-stimulated cells were analyzed as the ratio of CD4^+^/CD8^+^ T cells by FACS. Data illustrating representative experiments derived from quadruplicate experiments are shown as a graph (**c1**-**c2**) and the ratio of CD4^+^/CD8^+^ (**d**) (*t*_(4)_ = 3.422, *P* = 0.0267). The production of intracellular cytokines IL-4 and IFN-γ by CD4+ T cells was measured by FACS (**e**) IFN-γ: *t*_(3)_ = 15.67, *P* = 0.0006; IL-4: *t*_(3)_ = 2.031, *P* = 0.1352. Statistically significant differences compared with the PBS group are shown as *P < 0.05 or **P < 0.01

It has been shown in the RA vaccine mouse model that attenuated larvae stimulate proliferation of antigen-specific Th1 lymphocytes in the skin-draining lymph nodes. These CD4+ T cells can be recruited to the pulmonary parenchyma via the circulation and they provide the lungs with an immune micro-environment against a challenge with normal larvae [16, 17]. The production of IFN-γ and TNF-α by these cells is essential for cell-mediated protective immunity [18]. Therefore, we speculated that SjCRT could also induce a predominantly Th1 immune response in the mouse spleen. To test this hypothesis, we first detected the proliferation of splenic lymphocytes from sensitized mice in response to rSjCRT re-stimulation using the MTT method and found that the values of stimulation index in splenic lymphocytes in rSjCRT-stimulated and ConA groups were significantly higher than that in the PBS control group (Fig. 6a) (SjCRT: *t*_(3)_ = 20.44, *P* = 0.0003; ConA: *t*_(3)_ = 6.888, *P* = 0.0063). This indicated that SjCRT can enhance lymphocyte proliferation in the spleen from mice immunized with rSjCRT, and SjCRT-specific recall response might be elicited by rSjCRT re-stimulation. Then, we investigated the profiles of cytokines secreted by these cells. Splenocytes from sensitized mice were incubated with rSjCRT at 37 °C and 5% CO_2_ for 68 h, and the levels of cytokine production in the culture supernatant were determined by ELISA (Fig. 6b). The splenocytes of mice immunized with rSjCRT produced much higher levels of proinflammatory (TNF-α: *t*_(3)_ = 41.39, *P* < 0.0001 and IFN-γ: *t*_(3)_ = 29.63, *P* < 0.0001) and Th2-type (IL-4: *t*_(3)_ = 6.295, *P* = 0.0081) cytokines than the cells of the control mice, whereas there was no significant difference in the production of a regulatory cytokine IL-10 (*t*_(3)_ = 1.510, *P* = 0.2283) between the SjCRT-stimulated and control groups, suggesting that a Th1-biased immune microenvironment can be elicited by rSjCRT re-stimulation in the spleen from mice immunized with rSjCRT. Furthermore, we asked whether CD4+ T cells may also be involved in the above activity. To this end, we analyzed the ratio of CD4+ T / CD8+ T cells in the spleens of mice either immunized with rSjCRT or PBS-vaccinated using FACS. We found that the ratio value (equal to 2.26) of CD4+ T / CD8+ T cells from the splenocytes of rSjCRT-immunized mice was significantly higher than that (equal to 1.76) of CD4+ T / CD8+ T cells from control mice (*t*_(4)_ = 3.422, *P* = 0.0267) (Fig. 6c1-c2, d), indicating that CD4+ T cells are mobilized in the spleens from rSjCRT-immunized mice. Then, after splenocytes of rSjCRT/PBS-immunized mice were re-stimulated with rSjCRT/PBS, we detected production of the intracellular cytokines IFN-γ and IL-4 by CD4+ T cells from the above splenocytes using FACS. The results showed that CD4+ T cells from splenocytes of rSjCRT-immunized mice produced higher levels of IFN-γ than the cells from splenocytes of control (PBS-immunized) mice (Fig. 6e) (*t*_(3)_ = 15.67, *P* = 0.0006). In contrast, the level of IL-4 produced by CD4+ T cells from splenocytes of rSjCRT-immunized mice did not differ significantly from the cells of control mice (*t*_(3)_ = 2.031, *P* = 0.1352). These results indicated that IFN-γ-producing CD4+ T cells or Th1 cells were activated by rSjCRT re-stimulation and may contribute to the generation of a Th1-biased immune microenvironment in the spleens of rSjCRT-immunized mice.

## Discussion

The importance of calreticulin as an effector molecule of immunogenic cell death in radiation cancer therapy has been established in recent studies [4, 19]. In the present study, we concentrated on immunogenic properties of SjCRT from both RA schistosomula (RAS) and recombinant protein, and we provide preliminary evidence for the exposure of SjCRT on the surface of cells in early stage RA schistosomula and activation of immune cells (DC and lymphocytes) stimulated with rSjCRT protein in the mouse model. These findings suggest that SjCRT might be one of the main effectors of immunogenic cell death and may play an important role in protective immunity induced by the RA schistosomula vaccine.

The current consensus [3] is that a focal response around challenge larvae is cell-mediated in pulmonary parenchyma and this effector mechanism involves IFN-γ-producing CD4+ T cells, and TNF-α-mediated immune responses are required for this protective immunity. This suggests that a Th1 biased microenvironment is essential for immune responses in RA vaccine models. This type of immunity is similar to that of the immune response induced by intracellular protozoa [20], instead of a Th2-mediated protective immunity elicited by nematode helminths [21]. These proinflammatory Th1 responses are supposed to be elicited by RA schistosomula cells undergoing immunogenic cell death [22]. In fact, our preliminary studies demonstrated that different forms of cell death were observed by transmission electron microscopy (TEM) in cells from both normal and RA larvae. Thus, cells derived from RA schistosomula have been shown to have morphological features of necrotic cell death during the course of *in vitro* culture for 14 days, whereas normal larvae-derived cells have the characteristics of apoptotic cells (unpublished data). Therefore, we speculated that RAS-derived cells may have some properties of immunogenic cell death that is induced by certain agents (e.g. anthracyclines, irradiation and hypericin-based photodynamic therapy) in a tumor prophylactic vaccination model, in which dying cancer cells’ exposure or release of several molecules (e.g. calreticulin, HSP70, HMGB1 and ATP) dictate tumor immunogenicity, and these DAMPs are able to activate various kinds of immune cells such as natural killer cells (NK), macrophages and DCs [5]. To validate this hypothesis, we investigated the expression profile of SjCRT in cells from RA and normal schistosomula cultured *in vitro* at different points of time, and we found that intracellular SjCRT is expressed at higher levels in early stage RA schistosomula, suggesting that the increase in the abundance of intracellular SjCRT might be correlated with the early SjCRT exposure on the surface of the plasma membrane. This deduction is also supported by our previous observation in which SjCRT was located near the cell membrane within cytoplasmic areas of RAS-derived cells on day 7 after UV irradiation of schistosoma larvae. In contrast, SjCRT in the normal larvae-derived cells is distributed throughout the whole cytoplasm (unpublished data). This finding demonstrated that RAS-derived cells translocate relatively more SjCRT into the cell membrane than normal larvae-derived cells during early stages. As previously indicated [9], early-stage RAS-derived cells undergo a cellular stress response with characteristics of elevated SjHSP70 levels on the surface of the cells, and this response might inhibit cell apoptosis induced by UV-irradiation, causing necroptosis-like cell death of RA schistosomula. With extended culture time, the expression of SjCRT in cells from RA larvae was significantly lower than that from corresponding normal schistosomula. It is probable that damage of cells treated with UV irradiation is more severe than of normal cells cultured *in vitro*; this might lead to a breach of plasma membrane integrity and decrease the levels of cytoplasmic and membrane-bound SjCRT. Therefore, in terms of exposure/release of SjCRT and SjHSP70 molecules, the form of cell death in RA schistosomula is different from that in normal larvae. Thus, a plausible explanation is that cells from normal schistosomula cultured *in vitro* may have more apoptotic properties, whereas cells from RA parasites may show more necroptotic features. These different forms of cell death lead to distinct types of immune responses. When these apoptotic cells are engulfed by dendritic cells they may bring about a tolerance in hosts, whereas necroptotic cells may elicit an immunogenic response [23, 24]. Whether it is truly the case remains to be determined experimentally.

As we have known, CRT as one of the ‘eat me’ signal molecules plays a crucial role in the clearance of dying cells [6]. Generally speaking, tolerogenic ‘eat me’ signals that interact with immature DCs might cause tolerogenic phagocytosis whereas immunogenic ‘eat me’ signals that interact with immature DCs might favor immunogenic phagocytosis. DCs that undergo tolerogenic phagocytosis are unable to attain functional maturation, whereas DCs that undergo immunogenic phagocytosis can exhibit phenotypic and functional maturation; namely, they can present antigens to CD4+ T cells under the condition of elevated levels of co-stimulatory molecules and proinflammatory cytokines [25]. Many studies have indicated that exposure and release of CRT could induce DC maturation, activation and related cytokine secretion [26]. Therefore, we speculated that SjCRT has similar functions. To this end, we first obtained recombinant SjCRT protein (rSjCRT), and we then investigated whether rSjCRT is able to elicit activation and maturation of mouse DCs. We found that rSjCRT indeed could elicit phenotypic and functional maturation of DCs, especially producing higher levels of proinflammatory cytokines (TNF-α and IFN-γ), indicating that SjCRT can mediate immunogenic phagocytosis by mouse DCs and might provide a microenvironment for CD4+ T-cell-mediated adaptive immunity induced by RA schistosomula vaccines. We also found that the aforementioned immunogenic phagocytosis might be mediated by CD91 on the surface of DCs. This is not unexpected, since CD91 was previously reported to be a common receptor of heat-shock/chaperone proteins (gp96, Hsp70, Hsp90 and CRT), and this receptor is responsible for the cross-presentation of HSP-chaperoned peptides, leading to priming of T cell responses, especially peptide-specific cytotoxic T lymphocyte response in anticancer immunity [12]. Furthermore, several recent studies have found that CD91 might be involved in mediating CD4+ T cell responses; CD91 may take part in Hsp70-facilitated activation of human antigen-specific CD4+ memory T cells [27], and CD91 may also serve as a signaling receptor for these immunogenic HSPs, resulting in the maturation of DCs, cytokine secretion and priming of T-helper (Th) cells [28]. These data suggest that CD91 may be both an endocytic receptor and a signaling receptor to be engaged upon activation of DCs and differentiation of CD4+ T cells. In this study, we also confirmed that mDCs pulsed with rSjCRT can polarize naive CD4+ T cells towards differentiation of Th1 cells *in vitro*. A plausible explanation is that CD91 on the surface of mouse DCs might bind to SjCRT, allowing for internalization of SjCRT, regulating cell signaling for DC activation and secretion of proinflammatory cytokines (TNF-α and IFN-γ). This cytokine pattern is likely to be able to direct T cell responses to a Th1 phenotype. However, the mechanism underlying this process remains to be further elucidated experimentally.

Previous studies have demonstrated that the prolonged residence of RA schistosomula in the skin-draining lymph nodes (SLN) stimulates intense proliferation of antigen-specific Th1 lymphocytes, and these CD4+ T cells with Th1 characteristics can be recruited to the pulmonary parenchyma via the circulation. They may provide the lungs with an immune micro-environment against a challenge with normal larvae, suggesting that aforementioned CD4+ Th1 cells are mainly generated in the SLN [17, 19]. However, they are also likely to be generated in the spleens of mice vaccinated with attenuated larvae because very little parasite-released material may be present in this organ due to the death of cells from attenuated larvae. In fact, it was reported that a high level of IFN-γ can be detected in the mouse spleen in response to soluble schistosomular protein following vaccination with attenuated larvae [17], suggesting that an antigen-specific Th1 immune response can be elicited in the spleen. In the present study, we found that rSjCRT can stimulate lymphocyte proliferation in the spleen of mice immunized with rSjCRT, and CD4+ T cells from splenocytes of rSjCRT-immunized mice produced much higher levels of IFN-γ. These data further support the notion that SjCRT can also induce Th1-biased immune responses in the mouse spleen. We believe the immune property of these memory IFN-γ-producing CD4+ T cells is similar to that of the cells generated in SLN, namely that these CD4+ T cells might also be recruited to the lungs and provide an effector cell population that can be rapidly stimulated by the challenge parasites [17]. However, the role of SjCRT in protective immunity induced by RA schistosomula remains to be demonstrated *in vivo*.

## Conclusions

Our data suggest that SjCRT can be exposed on the surface of cells in the early-stage RA schistosomula, and rSjCRT can activate mouse DCs in a CD91-dependent manner and induce a Th1-polarized immune response in mice. Therefore, SjCRT can be considered a main effector of immunogenic cell death and might play a role in conferring a Th1-polarized immune response induced by RA cercariae/schistosomula in mice.

## Abbreviations

APC: Antigen present cells
BSA: Bovine serum albumin
CCR7: C-C chemokine receptor type 7
ConA: Concanavalin A
CRT: Calreticulin
CXCR4: C-X-C chemokine receptor type 4
DAMPs: Damage-associated molecular patterns
DC: Dendritic cells
DMSO: Dimethyl sulfoxide
ELISA: Enzyme-linked immunosorbent assay
GM-CSF: Granulocyte-macrophage colony stimulating factor
HSP: Heat-shock protein
ICD: Immunogenic cell death
IFN: Interferon
IL: Interlukine
LPS: Lipopolysaccharide
mAbs: Monoclonal antibodies
MFI: Mean fluorescence intensity
MTT: 3-(4,5-dimethyl-2-thiazolyl)-2,5-diphenyl-2-H-tetrazolium bromide
PMA: Phorbol-12-myristate-13-acetate
RA: Radiation-attenuated
Sj: *Schistosoma japonicum*
TNF: Tumor necrosis factor
α2-M: α2-macroglobulin
